# Neutrophil-Lymphocyte Ratio as a Prognostic Parameter in NSCLC Patients Receiving EGFR-TKIs: A Systematic Review and Meta-Analysis

**DOI:** 10.1155/2021/6688346

**Published:** 2021-01-20

**Authors:** Mingbo Tang, Xinliang Gao, He Sun, Suyan Tian, Junxue Dong, Zhao Liu, Wei Liu

**Affiliations:** ^1^Department of Thoracic Surgery, The First Hospital of Jilin University, Changchun 130021, Jilin, China; ^2^Department of Division of Clinical Research, The First Hospital of Jilin University, Changchun 130021, Jilin, China; ^3^Department of Molecular Biology, Max Planck Institute for Infection Biology, Berlin 10117, Germany

## Abstract

**Objective:**

To research the impact of neutrophil-lymphocyte ratio (NLR) as a prognostic parameter in non-small-cell lung cancer (NSCLC) patients treated with epidermal growth factor receptor tyrosine kinase inhibitors (EGFR-TKIs).

**Methods:**

We searched the databases such as the American Society of Clinical Oncology (ASCO), EMBASE, PubMed, the European Society of Medical Oncology (ESMO), Wanfang, and CNKI for articles illustrating the impact of pretreatment NLR on survival data in NSCLC patients undergoing EGFR-TKIs treatment. We did a meta-analysis for overall survival (OS) and progression-free survival (PFS).

**Results:**

We recruited 10 studies in our meta-analysis. Our study suggested that patients with low NLR had better PFS (hazard ratio (HR) = 1.67, 95% confidence interval (CI) = (1.16–2.39), and *P* value = 0.005) and OS (HR = 1.66, 95% CI = (1.08–2.55), and *P* value = 0.02) in comparison to patients with high NLR.

**Conclusion:**

In conclusion, our meta-analysis revealed that lower NLR predicted a better survival (PFS and OS) in patients receiving the treatment of EGFR-TKIs.

## 1. Background

Lung cancer is among the leading causes of cancer death among both genders, with one-quarter of cancer death due to lung cancer [1]. Non-small-lung cancer (NSCLC) takes about 85% of all lung cancer types. Over 60% of patients with NSCLC had an expression of epidermal growth factor receptor (EGFR) [2]. Thus, EGFR has been a very vital treatment target for these NSCLC patients, which is more often detected in females and nonsmokers [3]. Tyrosine kinase inhibitors (TKIs) are important treatment targets for patients harboring activating mutations in the tyrosine kinase domains of the EGFR gene. Many studies identified the prognostic biomarkers for NSCLC patients undergoing EGFR-TKIs treatment, and neutrophil-to-lymphocyte ratio (NLR) has been an interesting field. The NLR is calculated with absolute neutrophil counts divided by the absolute lymphocyte counts of a full blood count, and thus, the test cost of this biomarker is not expensive. NLR can be used as an inflammatory biomarker that indicates systematic inflammation [4]. Inflammation has a fundamental function in the tumor development and progress of cancer cells including proliferation, angiogenesis, and metastasis. Thus, NLR could serve as a prognostic factor. An Italian study consisting of 63 end-stage NSCLC patients with EGFR mutation treated with EGFR-TKIs suggested that patients with NLR lower than 3.5 had longer progression-free survival (PFS) and overall survival (OS) in comparison with those with NLR higher than 3.5 (PFS: hazard ratio (HR) = 2.275, *P* value = 0.007; OS: HR = 2.699, *P* value = 0.018) [5]. Another retrospective Japanese study recruiting 205 stage IV NSCLC patients under EGFR-TKIs treatment suggested that patients with NLR lower than 3.55 had longer PFS and OS compared with those with NLR higher than 3.55 (for PFS: HR = 1.82, *P* value <0.0001; for OS: HR = 1.78, *P* value <0.001) [6]. However, studies did not reach consistency [7, 8].

The previous meta-analysis revealed that NLR predicted elevated length of survival in NSCLC patients with systematic treatment including chemotherapy, targeted therapy, and immunotherapy [9, 10]. However, recruiting clinical studies with different treatment methods and patients in different stages increased the heterogeneity of our study. No meta-analysis focused on the impact of NLR on prognosis in NSCLC patients receiving EGFR-TKIs. Thus, we decided to do a meta-analysis investigating clinical studies about pretreatment NLR level on survival in NSCLC patients with EGFR-TKIs treatment. Our hypothesis is that patients with lower NLR could be a prognostic parameter for improved length of survival in patients with NSCLC treated with EGFR-TKIs.

## 2. Methods

### 2.1. Literature Search

The American Society of Clinical Oncology (ASCO), EMBASE, PubMed, European Society of Medical Oncology (ESMO), Wanfang, and CNKI databases were searched by independent researchers with the following keywords: non-small cell lung cancer, lung cancer, neutrophil lymphocyte ratio, epidermal growth factor receptor tyrosine kinase inhibitors, NSCLC, NLR, EGFR-TKIs, erlotinib, gefitinib, icotinib, afatinib, osimertinib, survival, PFS, and OS. We followed the methods of Xu et al. [11]. Two researchers searched the database for all published papers.

### 2.2. Inclusion Criteria

We selected published articles meeting all the following criteria: (1) clinical trials of patients who were cytologically or pathologically diagnosed with NSCLC and received EGFR-TKIs; (2) the clinical trials evaluated the length of survival data, including PFS and OS with a HR and also 95% confidence interval (CI).

### 2.3. Extraction of Study Results

Two independent medical doctors in our department read and approved all the papers independently and finally reached a consensus about the inclusion of the studies. When they cannot reach a consensus, a third researcher took part in the study inclusion procedures. We used the criteria defined by Cochrane Handbook for Systematic Reviews of Interventions version 5.1.0 [12], which is commonly used for meta-analysis. We summarized the study characteristics including the name of the first author, the publication time, country information, number of patients, study design, NLR cutoff values, treatment, median PFS, median OS, and follow-up time (Table 1).

### 2.4. Meta-Analysis

PFS and OS were chosen as the primary endpoints of our systematic meta-analysis. The PFS and OS correlated with NLR are summarized in Table 1. We calculated HR with 95% CI as indicators of prognosis with Review Manager (RevMan) version 5.4. Publication bias was calculated using Begg's and Egger's tests and funnel plot. We used the chi-square test and the *I*^2^ statistic to evaluate the statistical heterogeneity. An *I*^2^ value >50% was considered to suggest a heterogeneity of various studies. When significant heterogeneity was detected, a random-effects model was conducted. An *I*^2^ below 50% means no significant heterogeneity between these study results, and thus, a fixed-effects model was conducted.

## 3. Results

### 3.1. Study Characteristics of the Recruited Studies

**Figure 1:**
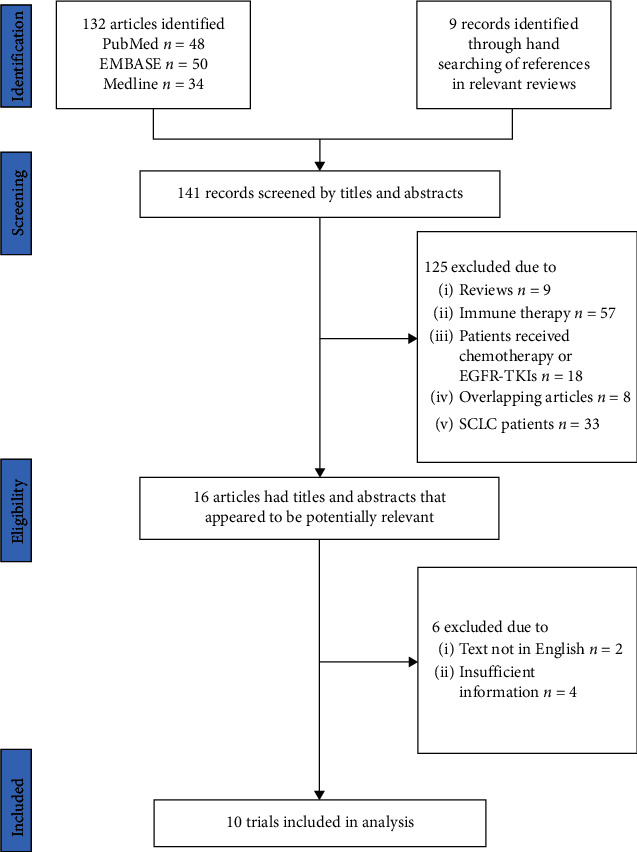
Study flow diagram.

In total, 10 clinical studies met the inclusion criteria and thus were included in our meta-analysis, with 9 trials about the significant relevance of NLR on PFS and 7 trials about the significant relevance of NLR on OS. The study flow diagram is illustrated in Figure 1. The study characteristics of the ten recruited articles are summarized in Table 1, including author's name, publication year, patient source (country), number of patients, study design, NLR cutoff values, treatment, median PFS, median OS, and follow-up time. All 10 studies met the allocation concealment.

### 3.2. Meta-Analysis regarding the Prognostic Relevance of NLR on PFS

**Figure 2:**
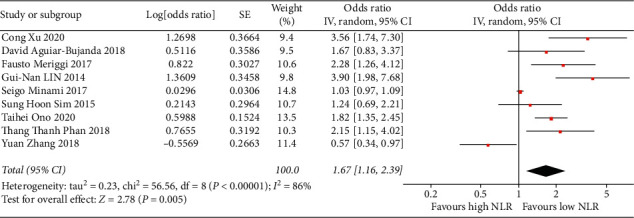
Meta-analysis of the impact of NLR on PFS in NSCLC patients with the treatment of EGFR-TKIs.

We recruited 9 clinical trials [5–8, 13–17] including 931 NSCLC patients and investigated the comparison of PFS among patients with low NLR versus patients with high NLR. Our meta-analysis indicated that patients with low NLR had better PFS compared with patients with high NLR (HR = 1.67, 95% CI = (1.16–2.39), and *P* value = 0.005, Figure 2).

### 3.3. Meta-Analysis regarding the Prognostic Relevance of NLR on OS

**Figure 3:**
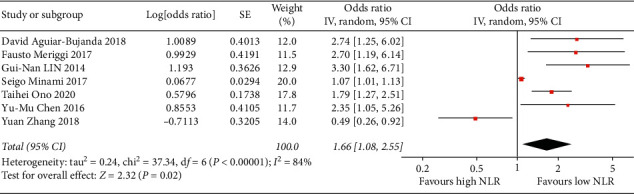
Meta-analysis of the impact of NLR on OS in NSCLC patients with the treatment of EGFR-TKIs.

We recruited 7 clinical trials [5–8, 15, 17, 18] including 2055 NSCLC patients and investigated comparison of OS among patients with low NLR versus patients with high NLR. Our meta-analysis indicated that patients with low NLR had better OS compared with patients with high NLR (HR, 1.66, 95% CI = (1.08–2.55), and *P* value = 0.02, Figure 3).

### 3.4. Publication Bias

No publication bias was detected in our meta-analysis using funnel plot, Egger's test, and Begg's test (all *P* values >0.05).

## 4. Discussion

Our study suggested that NLR could serve as a prognostic factor for PFS and OS in NSCLC patients undergoing EGFR-TKIs treatment. NLR is calculated as the ratio of circulating neutrophil to lymphocyte counts. Neutrophils serve as especially important cells in inflammatory response. Furthermore, neutrophils, the most abundant type of leukocytes in blood accounting for 50–70% of all leukocytes, with a nearly seven-hour half-life in healthy people [19], specifically support the initiation of metastasis [20]. A study using mouse breast cancer model suggested that neutrophil-derived leukocytes support the colonization of cancer cells with a higher tendency of metastasis [20]. Neutrophils expand both in the tumor microenvironment and throughout the body, which in tumor-bearing hosts can oppose or potentiate the progression of cancer cells. These two types of neutrophil behavior are regulated by signaling pathways regulated in the tumor microenvironment by tumor cells or stromal cells, which have the function of educating neutrophils to execute the death of the tumor or support tumor spread [21]. Patients with several cancer types, including but not limited to lung cancer, always have a higher number of circulating neutrophils [22]. Animal studies suggested that noncirculating neutrophils are retained longer time in tumor tissues compared to the time in the spleen, indicating that tumor microenvironment encourages the survival of neutrophils [23]. Evidence suggested that circulating neutrophils had a half-life in cancer patients as long as 17 hours [24]. A longer half-life indicated that neutrophils have more time to perform the carcinogenesis during tumor development. The role of neutrophils in tumor development can be regulated by tumor growth factor-*β* (TGF-*β*). A mouse model of subcutaneous mesothelioma tumors treated with a TGF-*β* inhibitor proved that neutrophils support the growth of tumor cells by inhibiting CD8^+^ T cells in the untreated group, while opposed the tumor growth through cytotoxic ability in the TGF-*β*-treated group. Neutrophils work as a link between inflammation and cancer and have a tumor growth-promoting effect [25]. NLR had a prognostic role in other cancers [26]. In patients with hepatocellular carcinoma (HCC) receiving sorafenib, NLR also showed a prognostic role. Patients with NLR higher than 3 had a lower median PFS compared with those with NLR lower than 3 (2.6 vs. 3.3 months, *P* value <0.049), but no significant difference was observed regarding median OS [27]. Our study also supported that patients with low NLR had better survival data in NSCLC patients undergoing EGFR-TKIs treatment, indicating neutrophil as a negative prognostic parameter in cancer patients.

Lymphocyte keeps a vital role in tumor development for mediating anticancer immunity. There are two main types of lymphocytes, B lymphocytes and T lymphocytes. T lymphocytes are defined by the expressions of T-cell receptors (TCRs) for recognizing antigens. There are two types of T cells, which are CD4^+^ T helper cells (T_H_) and CD8^+^ cytotoxic lymphocytes (CTL). CD8^+^ CTLs are critical mediators in the antitumor immunity due to their function to directly kill cancer cells [28]. CD8^+^ CTLs could produce interferon-*γ* (IFN-*γ*), which could enhance their ability to motility, particularly speed and also its cytotoxic function [29]. Studies have shown a positive association between increased CD8^+^ CTLs in the tumor microenvironment and better prognosis in cancer patients, including but not limited to cervical cancer patients, breast cancer patients, and colorectal cancer patients [30–32]. Thus, an elevated CD8^+^ CTLs could link to a better prognosis in many cancer types. CD4^+^ T cells enhance the antitumor immunity by providing help for CD8^+^ CTLs and antibody responses, together with the help of secretion of the interferon-gamma (IFN-*γ*) and tumor necrosis factor-*α* (TNF-*α*) [33]. Our study indicated the same results to previous studies, proving that higher NLR links to better prognosis in NSCLC patients.

Previous studies indicate that, for NSCLC patients receiving immunotherapy, pretreatment NLR serves as a prognostic factor [34]. Our study focused on the NSCLC patients undergoing EGFR-TKIs since more than 60% of NSCLC patients express the EGFR gene and could be candidates for EGFR-TKIs treatment. It is of great significance to illustrate the prognostic factor for this cohort of patients. Thus, our study was the first to prove the association of lower NLR with better PFS (HR = 1.67, 95% CI = (1.16–2.39), and *P* value = 0.005) and OS (HR = 1.66, 95% CI = (1.08–2.55), and *P* value = 0.02) in NSCLC patients treated with EGFR-TKIs.

Our study also has limitations. The studies were recruited using various cutoff points of NLR, ranging from 2.11 to 5.2. The reason might be different studies using the best cutoff point that differentiated the patients that could gain survival benefit. This increased the heterogeneity of our study and raised the difficulty of this biomarker to be used in the clinic. Thus, a large-scale clinical trial is needed to define a cutoff point of the NLR that could be used in the clinic. Nevertheless, our study showed the tendency of worse survival in NSCLC patients with higher pretreatment NLR levels. NLR, as a reasonable and not expensive biomarker, could be used as a clinical routine in NSCLC patients.

## 5. Conclusion

In conclusion, our meta-analysis revealed that lower NLR predicted better PFS and OS in NSCLC patients receiving EGFR-TKIs.

## Figures and Tables

**Table 1:** Study characteristics of studies investigating the prognostic relevance of NLR.

Author	Year	Country	Total number of patients	Study design	Clinical stage	NLR cutoff value	Treatment	Median PFS	Median OS	Follow-up time (months)
Thang Thanh Phan	2018	Vietnam	112	Single-center, retrospective study	IV	2.96	Erlotinib or gefitinib	11.1 vs. 7.7 months, HR = 2.15, 95% CI = (1.15–3.99), *P*=0.016	NA	NA
Sung Hoon Sim	2015	Korea	85	Single-center, retrospective study	IIIB or IV	3	Gefitinib	HR = 1.239, 95% CI = (0.693–2.215), *P*=0.469	NA	NA
Seigo Minami	2017	Japan	152	Single-center, retrospective study	Stage III-IV or postsurgical recurrence	2.11	Gefitinib, erlotinib or afatinib	15.9 vs. 10.1 months, HR = 1.03, 95% CI = (0.97–1.10), *P*=0.29	38.6 vs. 24.1 months, HR = 1.07, 95% CI = (1.01–1.14), *P*=0.03	NA
Fausto Meriggi	2017	Italy	63	Multicenter, retrospective study	IV	3.5	Erlotinib or gefitinib	HR = 2.275, 95% CI = (1.257–4.116), *P*=0.007	HR = 2.699, 95% CI = (1.187–6.137), *P*=0.018	NA
Yuan Zhang	2018	China	127	Single-center, retrospective study	IIIB or IV	2.9	Erlotinib or gefitinib	HR = 0.573, 95% CI = (0.340–0.964), *P*=0.036	HR = 0.491, 95% CI = (0.262–0.920), *P*=0.026	Mean: 28.12
Cong Xu	2020	China	65	Single-center, retrospective study	IIIB to IVB	2.57	Gefitinib, icotinib, erlotinib, afatinib, osimertinib	HR = 3.560, 95% CI = (1.736–7.301), *P* < 0.001	NA	NA
Yu-Mu Chen	2016	Taiwan, China	1386	Single-center, retrospective study	IIIB or IV	5.2	Erlotinib, gefitinib or afatinib	NA	HR = 2.352, 95% CI = (1.052–5.256), *P*=0.037	Median: 7.0
Gui-Nan LIN	2014	China	81	Single-center, retrospective study	IV	3.5	Erlotinib or gefitinib	HR = 3.89, 95% CI = (1.98–7.68), *P* < 0.001	HR = 3.29, 95% CI = (1.62–6.71), *P* < 0.001	NA
Taihei Ono	2020	Japan	205	Single-center, retrospective study	IV	3.55	Erlotinib or gefitinib	HR = 1.82, 95% CI = (1.35–2.44), *P* < 0.0001	HR = 1.78, 95% CI = (1.27–2.51), *P* < 0.001	Median: 25.2
David Aguiar-Bujanda	2018	Spain	41	Single-center, retrospective study	IIIB to IV	4.39	Erlotinib or gefitinib	10.58 vs. 20.84 months, HR = 1.668, 95% CI = (0.826–3.368), *P*=0.155	7.4 months vs. 24.6 months, HR = 2.743, 95% CI = (1.249–6.022), *P*=0.0123	Median: 54.5

NLR: neutrophil-lymphocyte ratio; HR: hazard ratio; CI: confidence interval; NA: not available; PFS: progression-free survival; OS: overall survival.

## Data Availability

All data generated or analyzed during this study are included in this published article.

## References

[1] Siegel R. L., Miller K. D., Jemal A. (2019). Cancer statistics, 2019. *CA: A Cancer Journal for Clinicians*.

[2] da Cunha Santos G., Shepherd F. A., Tsao M. S. (2011). EGFR mutations and lung cancer. *Annual Review of Pathology: Mechanisms of Disease*.

[3] Karachaliou N., Fernandez-Bruno M., Bracht J. W. P., Rosell R. (2018). EGFR first- and second-generation TKIs-there is still place for them in EGFR-mutant NSCLC patients. *Translational Cancer Research*.

[4] Martins E. C., Silveira L. D. F., Viegas K. (2019). Neutrophil-lymphocyte ratio in the early diagnosis of sepsis in an intensive care unit: a case-control study. *Revista Brasileira de Terapia Intensiva*.

[5] Meriggi F., Codignola C., Beretta G. D. (2017). Significance of neutrophil-to-lymphocyte ratio in western advanced EGFR-mutated non-small cell lung cancer receiving a targeted therapy. *Tumori Journal*.

[6] Ono T., Igawa S., Kurahayashi S. (2020). Impact of neutrophil-to-lymphocyte ratio in patients with EGFR-mutant NSCLC treated with tyrosine kinase inhibitors. *Investigational New Drugs*.

[7] Aguiar-Bujanda D., Dueñas-Comino A., Saura-Grau S. (2018). Neutrophil to lymphocyte ratio as a prognostic factor in European patients with epidermal growth factor receptor-mutant non-small cell lung cancer treated with tyrosine kinase inhibitors. *Oncology Research and Treatment*.

[8] Minami S., Ogata Y., Ihara S., Yamamoto S., Komuta K. (2017). Neutrophil-to-lymphocyte ratio predicts overall survival of advanced non-small cell lung cancer harboring mutant epidermal growth factor receptor. *World Journal of Oncology*.

[9] Wang Z., Zhan P., Lv Y. (2019). Prognostic role of pretreatment neutrophil-to-lymphocyte ratio in non-small cell lung cancer patients treated with systemic therapy: a meta-analysis. *Translational Lung Cancer Research*.

[10] Zhao Q. T., Yang Y., Xu S. (2015). Prognostic role of neutrophil to lymphocyte ratio in lung cancers: a meta-analysis including 7054 patients. *OncoTargets and Therapy*.

[11] Xu Y., Qiu Y., Yuan S., Wang H. (2020). Prognostic implication of human papillomavirus types in cervical cancer patients: a systematic review and meta-analysis. *Infectious Agents and Cancer*.

[12] Chien T. J., Hsu C. H., Liu C. Y., Fang C. J. (2017). Effect of acupuncture on hot flush and menopause symptoms in breast cancer- a systematic review and meta-analysis. *PLoS One*.

[13] Phan T. T., Ho T. T., Nguyen H. T., Nguyen H., Tran T. B., Nguyen S. T. (2018). The prognostic impact of neutrophil to lymphocyte ratio in advanced non-small-cell lung cancer patients treated with EGFR TKI. *International Journal of General Medicine*.

[14] Sim S. H., Beom S. H., Ahn Y. O. (2016). Pretreatment neutrophil‐lymphocyte ratio is not a significant prognostic factor in epidermal growth factor receptor‐mutant non‐small cell lung cancer patients treated with tyrosine kinase inhibitors. *Thoracic Cancer*.

[15] Zhang Y., Feng Y. C., Zhu H. G (2018). The peripheral blood neutrophil-to-lymphocyte ratio is a prognostic predictor for survival of EGFR-mutant nonsmall cell lung cancer patients treated with EGFR-TKIs. *Medicine*.

[16] Xu C., Yao X., Li T. (2020). Pretreatment neutrophil-to-lymphocyte ratio is a predictive biomarker for EGFR TKI-treated patients with advanced EGFR- mutant non-small cell lung cancer. *Translational Cancer Research*.

[17] Lin G.-N., Peng J.-W., Liu P.-P., Liu D.-Y., Xiao J.-J., Chen X.-Q. (2017). Elevated neutrophil-to-lymphocyte ratio predicts poor outcome in patients with advanced non-small-cell lung cancer receiving first-line gefitinib or erlotinib treatment. *Asia-Pacific Journal of Clinical Oncology*.

[18] Chen Y. M., Lai C. H., Rau K. M. (2016). Impact of clinical parameters and systemic inflammatory status on epidermal growth factor receptor-mutant non-small cell lung cancer patients readministration with epidermal growth factor receptor tyrosine kinase inhibitors. *BMC Cancer*.

[19] Saverymuttu S. H., Peters A. M., Keshavarzian A., Reavy H. J., Lavender J. P. (1985). The kinetics of ^111^indium distribution following injection of ^111^indium labelled autologous granulocytes in man. *British Journal of Haematology*.

[20] Wculek S. K., Malanchi I. (2015). Neutrophils support lung colonization of metastasis-initiating breast cancer cells. *Nature*.

[21] Coffelt S. B., Wellenstein M. D., de Visser K. E. (2016). Neutrophils in cancer: neutral no more. *Nature Reviews Cancer*.

[22] Templeton A. J., McNamara M. G., Šeruga B. (2014). Prognostic role of neutrophil-to-lymphocyte ratio in solid tumors: a systematic review and meta-analysis. *JNCI: Journal of the National Cancer Institute*.

[23] Sawanobori Y., Ueha S., Kurachi M. (2008). Chemokine-mediated rapid turnover of myeloid-derived suppressor cells in tumor-bearing mice. *Blood*.

[24] Steinbach K. H., Schick P., Trepel F. (1979). Estimation of kinetic parameters of neutrophilic, eosinophilic, and basophilic granulocytes in human blood. *Blut*.

[25] Pekarek L. A., Starr B. A., Toledano A. Y., Schreiber H. (1995). Inhibition of tumor growth by elimination of granulocytes. *Journal of Experimental Medicine*.

[26] Neuzillet C., Casadei Gardini A., Brieau B. (2019). Prediction of survival with second-line therapy in biliary tract cancer: actualisation of the AGEO CT2BIL cohort and European multicentre validations. *European Journal of Cancer*.

[27] Brunetti O., Gnoni A., Licchetta A. (2019). Predictive and prognostic factors in HCC patients treated with sorafenib. *Medicina*.

[28] Dunn G. P., Old L. J., Schreiber R. D. (2004). The three Es of cancer immunoediting. *Annual Review of Immunology*.

[29] Bhat P., Leggatt G., Waterhouse N., Frazer I. H. (2017). Interferon-*γ* derived from cytotoxic lymphocytes directly enhances their motility and cytotoxicity. *Cell Death & Disease*.

[30] Kim P. S., Ahmed R. (2010). Features of responding T cells in cancer and chronic infection. *Current Opinion in Immunology*.

[31] Kmiecik J., Poli A., Brons N. H. (2013). Elevated CD3^+^ and CD8^+^ tumor-infiltrating immune cells correlate with prolonged survival in glioblastoma patients despite integrated immunosuppressive mechanisms in the tumor microenvironment and at the systemic level. *Journal of Neuroimmunology*.

[32] Piersma S. J., Jordanova E. S., van Poelgeest M. I. E. (2007). High number of intraepithelial CD8^+^ tumor-infiltrating lymphocytes is associated with the absence of lymph node metastases in patients with large early-stage cervical cancer. *Cancer Research*.

[33] Tay R. E., Richardson E. K., Toh H. C. (2020). Revisiting the role of CD4(+) T cells in cancer immunotherapy-new insights into old paradigms. *Cancer Gene Therapy*.

[34] Zhang N., Jiang J., Tang S., Sun G. (2020). Predictive value of neutrophil-lymphocyte ratio and platelet-lymphocyte ratio in non-small cell lung cancer patients treated with immune checkpoint inhibitors: a meta-analysis. *International Immunopharmacology*.

