# Correction to “Neutrophil‐Lymphocyte Ratio as a Prognostic Parameter in NSCLC Patients Receiving EGFR‐TKIs: A Systematic Review and Meta‐Analysis”

**DOI:** 10.1155/jo/9826802

**Published:** 2026-09-06

M. Tang, X. Gao, H. Sun, et al., “Neutrophil‐Lymphocyte Ratio as a Prognostic Parameter in NSCLC Patients Receiving EGFR‐TKIs: A Systematic Review and Meta‐Analysis,” *Journal of Oncology*, 2021, no. 1 (2021): 1–7, https://doi.org/10.1155/2021/6688346.

In the article titled “Neutrophil‐Lymphocyte Ratio as a Prognostic Parameter in NSCLC Patients Receiving EGFR‐TKIs: A Systematic Review and Meta‐Analysis,” errors are present in Figures 2 and 3. Specifically, the data from Yuan Zhang 2018 [1] were presented incorrectly, showing progression‐free survival (PFS) and overall survival (OS) favour high neutrophil‐lymphocyte ratios (NLR), when the opposite was reported in the cited article.

**FIGURE 2:**
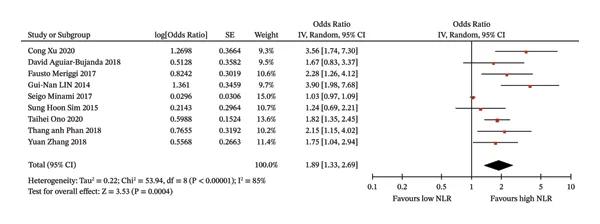
Meta‐analysis of the impact of NLR on PFS in NSCLC patients with the treatment of EGFR‐TKIs.

**FIGURE 3:**
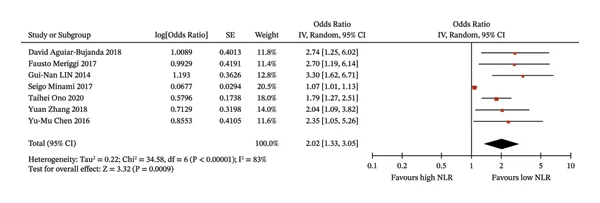
Meta‐analysis of the impact of NLR on OS in NSCLC patients with the treatment of EGFR‐TKIs.

The authors have performed the analysis with the correct values and confirmed that the conclusion that a higher NLR is associated with poorer survival outcomes in NSCLC patients receiving EGFR‐TKIs remains unchanged.

Figures 2 and 3 have been revised to reflect the analyses performed on the correct values and are shown below:

We apologize for these errors.
